# Surveillance of cardiovascular disease risk factors in India: The need & scope

**DOI:** 10.4103/0971-5916.73420

**Published:** 2010-11

**Authors:** Bela Shah, Prashant Mathur

**Affiliations:** *Division of Non-communicable Diseases, Indian Council of Medical Research, New Delhi, India*

**Keywords:** Cardiovascular diseases, risk factor, surveillance

## Abstract

There is a rise of non-communicable diseases (NCDs) burden, which is causing increasing morbidity and premature mortality in developing countries. In 1990, cardiovascular diseases (CVD) accounted for 63 per cent of all deaths and India contributed to 17 per cent to the worldwide mortality. Several surveys conducted across the country over the past two decades have shown a rising prevalence of major risk factors for CVD in urban and rural populations. These surveys are limited by their generalisability to other parts of the country, and more was required to roll out of an action plan. There was lack of an organized national system for monitoring these risk factors over time so as to inform policy and programme for appropriate interventions. The Indian Council of Medical Research (ICMR) leveraged its research on NCD risk factor surveillance to the development of the national plan under the Integrated Disease Surveillance Project (IDSP) which will obtain State-based prevalence of selected risk factors. This review provides the scenario of CVD in India and the need for a surveillance system. By examining similar experiences globally, it outlines the scope of CVD surveillance in India.

## Introduction

The health care needs of the world’s population are likely to undergo dramatic changes due to the ongoing demographic transition. Non-communicable diseases (NCDs), such as diabetes, cancer, depression and heart disease, are rapidly replacing infectious diseases and malnutrition as the leading causes of disability and premature death. Eighty per cent of total deaths due to non-communicable diseases occur in the low income countries. Men and women are equally affected. Cancer, cardiovascular diseases (CVD) and diabetes are becoming of serious concern, accounting for 52 per cent of deaths and 38 per cent of disease burden in the WHO South East Asia Region (SEAR). With the current trends, the top five causes of disability adjusted life years (DALYs) lost in 2020 are likely to be ischaemic heart disease, unipolar major depression, road traffic injuries, cerebro-vascular diseases, and chronic obstructive lung disease^1^. It has been estimated that a 2 per cent reduction in chronic diseases death rates per year globally could result in saving about 36 million premature deaths by the year 2015^2^.

While mortality due to communicable diseases is decreasing, that for non-communicable diseases is rising at a very rapid pace. The health policy makers are faced with the burden of providing resources for the control and prevention of both the existing communicable diseases, and the increasing number of non-communicable diseases. This becomes difficult since the programmes for prevention and control of communicable diseases drain the meager resources. It is, therefore, not surprising that India has faced a serious handicap while planning and initiating programmes and activities to combat non-communicable diseases, including cardiovascular diseases.

Disease and risk factor surveillance involves a systematic collection, analysis and interpretation of data. Changes in population health behaviour are also monitored over time. These data are used to inform the public and decision-makers for planning and evaluating prevention and control programmes and designing health policy and legislation. This paper discusses the need and scope of cardiovascular disease risk factor surveillance in India.

## Cardiovascular disease risk factors and surveillance: the need

‘Risk’ is defined as a probability of an adverse health outcome, whereas ‘risk factor’ refers to an attribute or characteristic or exposure of an individual whose presence or absence raises the probability of an adverse outcome^3^. The World Health Report 2002 identifies top 20 leading risk factors in terms of the burden of disease according to the mortality status in the population^7^. Ezatti *et al*^4^ estimated that in 2000, 47 per cent of premature deaths and 39 per cent of total disease burden resulted from the combined effects of the risk factors studied.

The widely accepted concept of public health surveillance is the ongoing systematic collection, analysis and interpretation of health data essential for planning, implementing, and evaluating public health activities, closely integrated with timely dissemination of the data to enable effective and efficient action to be taken to prevent and control disease^5^. It ranges from compulsory notifiable diseases, specific disease registries (population-based, hospital-based), continuous or repeated surveys of representative samples of the population, to aggregate data for recording trends on consumption patterns and economic activity. It is important to differentiate surveys from surveillance as the former does not imply data collection for action.

The need for CVD surveillance arises from the demographic transition being accompanied by a “risk transition”. In the context of public health, population measurements of these risk factors are used to describe the distribution of future disease burden in a population, rather than predicting the health of a specific individual. Knowledge of risk factors can then be applied to shift population distributions of these factors. Information on disease occurrence is important in assisting health services planning, determining public health priorities, and monitoring the long term effectiveness of disease prevention activities. Thus, where resources permit, disease surveillance should also be included in the surveillance systems. Data collected from ongoing health information systems may be useful for surveillance when systematically analyzed and applied to policy in a timely manner. While surveys can be a one-off exercise, surveillance involves commitment to data collection on an ongoing (repeated, continuous) basis, as well use of the data for informing public health policies and programme. There are different aspects of ongoing versus periodic data collections that need to be considered in planning NCD surveillance. Nevertheless, regional surveys undertaken on a periodic basis are more often seen as easier to implement than large-scale national surveys.

Surveillance of cardiovascular diseases involves a lot of human and financial resources for its sustainability. Further focusing on disease results in identifying individuals at the downstream and potentially limits intervention. Risk factors are present for a long period of time during the natural history of CVD. It is now well established that a cluster of major risk factors (tobacco, alcohol, inappropriate diet, physical inactivity, obesity, hypertension, diabetes and dyslipidaemias) govern the occurrence of CVDs much before these are firmly established as diseases. Collecting data on these and monitoring their trends is a good beginning towards disease surveillance. It helps in making projections of trends of disease prevalence. Since these risk factors are amenable to interventions, efforts to tackle these would reduce the overall disease burden and promote health. Surveillance can be targeted at the entire population, at the high risk population, and special settings (workplace, schools, hospitals). At the local level, surveillance alerts the public health authorities on the trends and impact of interventions, at State level it helps in evaluating policy and making the necessary changes, while at the national level it helps in programme development and monitoring.

## Cardiovascular diseases in India

Cardiovascular diseases account for high morbidity and mortality all over the world. Countries where the epidemic began early are showing a decline due to major public health interventions. On the other hand, cardiovascular diseases are contributing towards an ever-increasing proportion of the non-communicable diseases in the developing countries^6^–^9^.

Cardiovascular diseases have assumed epidemic proportions in India as well. The Global Burden of Diseases (GBD) study reported the estimated mortality from coronary heart disease (CHD) in India at 1.6 million in the year 2000^10^. A total of nearly 64 million cases of CVD are likely in the year 2015, of which nearly 61 million would be CHD cases (the remaining would include stroke, rheumatic heart disease and congenital heart diseases). Deaths from this group of diseases are likely to amount to be a staggering 3.4 million^11^.

Coronary heart disease is more prevalent in Indian urban populations and there is a clear declining gradient in its prevalence from semi-urban to rural populations. Epidemiological studies show a sizeable burden of CHD in adult rural (3–5%) and urban (7–10%) populations. Thus, of the 30 million patients with CHD in India, there would be 14 million of whom are in urban and 16 million in rural areas. In India about 50 per cent of CHD-related deaths occur in people younger than 70 yr compared with only 22 per cent in the West. Extrapolation of these numbers estimates the burden of CHD in India to be more than 32 million patients^12^.

The ICMR-WHO study on Burden of Disease reviewed literature till 2003 on NCDs^13^. The weighted average prevalence for ischaemic heart disease was estimated to be 6.4 per cent in urban areas and 2.5 per cent in rural areas. The meta-analysis of eight studies carried out between 1995 and 2002 in urban areas gives a pooled prevalence rate of hypertension as 164 per thousand, and 157 per thousand in rural areas. The combined urban and rural pooled estimate of prevalence rate of hypertension among adults (>20 yr) was 159 per thousand. An increase of 17.5 per cent in the number of stroke cases in India occurred during the last one and a half decade. Mortality due to strokes has increased by 7.8 per cent from 1998 to 2004. Available evidence yielded that over 9 million stroke cases and about 6.4 million years have been lost due to disability during 2004^11^.

This increase in CVDs could be attributable to (*i*) increase in the population size due to natural growth, (*ii*) ageing of the population which makes people more vulnerable to chronic diseases at older ages, and (*iii*) increased vulnerability due to lifestyle changes.

## Cardiovascular disease surveillance: The international experience

Worldwide there are surveillance systems which have addressed the risk factors of CVD. The Behaviorial Risk Factor Surveillance Systems (BRFSS) was established in 1984 by the Centers for Disease Control (CDC, Atlanta, USA) as a state-based system of health surveys that collects information on health risk behaviours, preventive health practices, and health care access primarily related to chronic disease and injury^14^. Currently, data are collected monthly in all 50 States, the District of Columbia, Puerto Rico, the U.S. Virgin Islands, and Guam. More than 350,000 adults are interviewed each year, making the BRFSS the largest telephone health survey in the world. States use BRFSS data to identify emerging health problems, establish and track health objectives, and develop and evaluate public health policies and programmes. Many States also use BRFSS data to support health-related legislative efforts. All BRFSS States prepare reports for dissemination and education of the public, health professional community and State legislators on the prevailing health behaviour indicators. The landmark efforts done under the North Karelia Project in Finland since 1972 have demonstrated the usefulness of risk factor surveillance in evolving a public health response for the prevention and control of CVDs^15^. Repeated surveys assessed the impact of the community intervention programmes for risk factor reduction and thus helped in fine tuning the policies. It demonstrated benefits of developing linkages with important stakeholders. Till 1997 (25 yr), the smoking prevalence among men had declined from 52 to 31 per cent, whereas it increased in women from 10 to 16 per cent. During this period the all CVD related deaths declined by 68 per cent among men aged 30–59 yr^15^. The FINBALT Health Monitor is a collaborative system for monitoring health behaviours and related factors in Estonia, Finland, Latvia and Lithaunia. It serves public information, programme planning and evaluation needs of these individual countries. It also provides a data bank for researchers to work upon^16^. The World Health Organization (WHO) formulated the Global STEPwise approach for NCD risk factor surveillance aimed at collecting data on risk factors in a stepwise manner according to the complexities involved that would be comparable across variable sites in the world. It emphasizes on ‘core’, ‘expanded’ and ‘optional’ variables which provide a common platform for comparability and flexibility to include variables for local requirements^17^. Through the WHO efforts, Regional collaborations have been formed which aim at integrated prevention and control of NCDs and include risk factor surveillance as a major component: CINDI, CARMEN, SEANET, EMAN, NANDI and MOANA^18^.

Thus, the need for surveillance of CVDs and their risk factors is to:

- Recognize cases or their clusters so as to mount an appropriate response,
- Identify trends of diseases and their risk factors based on previously collected information,
- Monitor the effectiveness of intervention programs/policies,
- Map out the distribution of cases and risk factors and pick up regional and sub-group differences,
- Identify new research issues based on the findings of surveillance and to strengthen it, and
- Facilitate advocacy, policy guidance, prioritization of allocation of resources.

In order to fulfill the above aims, surveillance should:

- evaluate the existing system (public and private sectors) for its delivery and usage,
- identify and involve all stakeholders from the planning of policy to its implementation,
- begin by measuring modest parameters accurately, reliably and timely, and build up on success with time,
- be flexible, sensitive and adaptable to the changing needs and multiple users,
- develop task specific guidelines, and
- undertake training and periodic refresher sessions with the personnel involved in the programme.

## Risk factors for cardiovascular diseases: Indian scenario

Traditionally, risk factors for CVDs have been categorized as behavioural, anthropometric and biochemical. Several epidemiological studies conducted on the prevalence of CVD risk factors have indicated to an increasing trend^19^. These are studies which have been done at several locations across the country, in different time periods and using varying study methodologies.

A compilation of the profile of behavioural, anthropometric and biochemical risk factors as available from some of the recent literature has been compiled in Table I.

**Table I:** Profile of reported behavioral, anthropometric, biochemical risk factors for cardiovascular diseases in India

	Gupta *et al*^20^	Chow *et al*^21^	Prabhakaran *et al*^22^	Hazarika *et al*^23^	Gupta *et al*^24^	Kaur *et al*^25^	Thankappan *et al*^26^	Anand *et al*^27^	Mehan *et al*^28^	Mehan *et al*^29^	Mohan *et al*^30^	Nongkynrih *et al*^31^	
Site	Jaipur	Andhra Pradesh	Delhi	Dibrugarh	Jaipur	Chennai	Kumarokam	Ballabgarh	Baroda	Baroda	Chennai	Ballabgarh	
Year	2007	2007	2005	2004	2002	2007	2006	2007	2006	2006	2008		
No of subjects	1091	345	2122	3180	1800	2262	4955	2564	220	121	1167	1513	
Population studied	Urban,	Rural	Industry, men only	Rural	Urban	Industry	Rural	Urban	Industry	Urban	Industry	15– 64 yrs Urban Rural Periurban	
Tobacco smoking (%)	24.3	19.9	36.0	12.5	23.9	20.2	17.8	Men- 36.5 Women- 7.0	13.6	22.3	10.0		
Alcohol consumers (%)	-	-	-	36.4	-	34.8	13.4	Men-25.9 Women- 0.0	-	-	3.5		
Physical inactive37.8 (%)	11.3	-	-	25.5	10.7	-	Men- 14.8 Women- 55	17.3	74.4	3.9		
Overweight(%) 44.2	16.9	35	6	27.4	36.3	21.1	Men- 16 Women-21.9	37.7	29.8	60.2		
Obese (%)	16.5	4.4	3.3	0.9	-	6.9	-	Men-3.5 Women-20.6	4.1	-	-		
Increased waist circumference (%)	Men-21.6 Women-42.2	-	43	-	-	50.1	26.3	-	32.3	14.8	-		
Hypertension (%)	37.3	20.3	30	33.3	36.9	17.2	36.7	Men-17.2 Women-15.8	20.5	-	25.4		
Diabetes# (%)	12.3	3.7	15		12.5	3.4			6.8		-	Men	Women
												11.4	9.4
												3.9	1.6
												108	14.1
High Total cholesterol (%)	39.1	12.3	30.1		39.1	30.3			40.5			Men	Women
												44.3	44.3
												24.5	30.1
												25.3	32.9
High LDL cholesterol (%)	41.5	12.3	67.2		41.5	-			-			-	
Low HDL cholesterol (%)	55	87.2	33		55	-			-			Men	Women
												23.2	12.2
												31.0	22.4
												40.6	22.4

# - includes self reported and measured

These studies show that urban populations had higher prevalence of CVD risk factors as compared to rural populations. Risk factor prevalence from slum/peri-urban areas lay somewhere in between the urban and rural populations, but more inclined towards urban trends. Alcohol as a risk factor was reported by very few studies. Fruit and vegetable consumption of at least 5 servings per day was very low. Subjects studied in the industrial settings were more physically active than those in the free living populations. Overweight, obesity and central obesity were more in the urban than in rural populations. Women showed higher obesity prevalence than men. Hypertension in most studies was reported in more than 20 per cent subjects. Diabetes and hyperlipidaemia prevalence also followed similar patterns. The data from these studies lack comparability due to methodological variations.

## Surveillance for cardiovascular disease risk factors: The ICMR initiative in India

The ICMR conducted a multi-centric study at Ballabgarh (Haryana), Chennai (Tamil Nadu), Dibrugarh (Assam), Delhi, Nagpur (Maharashtra) and Thiruvananthapuram (Kerala) on risk factors for non-communicable diseases with WHO support (unpublished data). It was aimed at developing sentinel sites for NCD risk factor surveillance across the country as well as assessing the feasibility of adapting the WHO STEPS instrument for use in surveillance in the country. The sites and investigators were purposefully selected so as to include interest, expertise, institutional support and regional variability into the study design. The questionnaire was piloted and translated into the local languages by the selected investigators. A common study protocol was developed and the study was centrally co-ordinated at the Division of NCD, Indian Council of Medical Research (ICMR), New Delhi. A common training programme was conducted and monitoring visits were undertaken by an expert team for assessment of situation in the field area and providing technical support to the site teams. The behavioural and anthropometric risk factor study was done between 2003–2005 (Phase I) and in a sub-sample (20%) of Phase I participants, biochemical risk factors were estimated in 2005–2006 (Phase II). The study adapted the WHO STEPS approach, and the questionnaire was accordingly modified. The study participants included men and women aged 15–64 yr, residing in the selected urban, rural and slum areas (Dibrugarh site studied peri-urban population instead of slums and in Delhi, rural subjects were not studied). The sample size was calculated for the 10 yr age categories (15–24, 25–34, 35–44, 55–64 yr) for each sex and population, and the target was 250 subjects per cell. The results from all Centers were collated centrally at the co-ordinating unit at ICMR and analyzed. The final analysis for Phase I was done on 44491 subjects across all centers. In Phase II the final samples size available for analysis was 7876 subjects. The highlights of the results are summarized in Table II.

**Table II:** ICMR-who six site study: profile of reported behavioral, anthropometric and biochemical risk factors among men and women aged 15–64 yr in urban, rural and peri-urban/slum populations (unpublished data)

Characteristics	Men			Women		
	Urban (N=7557)	Rural (N=6668)	Slum/periurban(N=7646)	Urban (N=7666)	Rural (N=6849)	Slum/periurban (N=8105)
Daily tobacco smokers (%)	26.5	26.7	34.3	0.7	4.3	2.7
Mean age at initiation of smoking (yr) (SE)	21.6 (7.0)	21.96(8.0)	20.3 (7.5)	34.8 (12.3)	32.2 (11.8)	32.1 (13.5)
Daily smokeless tobacco consumption (%)	19.9	36.1	37	7.6	20	20.2
Ever consumption of alcohol (%)	40.1	49	54.7	3.4	8.3	13.7
Mean number of days/week of fruit consumption (SE)	2.9 (2.4)	1.5 (1.9)	1.9 (2.1)	2.7(2.3)	1.2 (1.6)	1.6 (1.8)
Mean number of days/week of vegetable consumption (SE)	5.8 (1.6)	5.6 (1.6)	5.1 (1.9)	5.8 (1.5)	5.5 (1.6)	5.2 (1.8)
Subjects consuming < 5 servings of fruits and vegetables per day (%)	79.2	82.1	85.1	84.1	87.2	90.2
*% Subjects reporting physical inactivity*						
Work	69.7	40.8	50.3	61	37	39.7
Transportation	37.1	12.9	17.9	46.3	29.6	34.4
leisure time	76.6	81	84	86.4	90.5	94.1
Mean BMI (kg/m^2^) (SE)	23.1 (0.5)	20.3 (0.4)	21.2 (0.4)	23.9 (0.6)	20.7 (0.5)	22.2 (0.5)
Proportion with BMI (%)	N- 7461	6613	7518	7556	6756	7952
25–29.9	25.5	8.3	14.4	27.7	11.5	19.6
≥30	5.4	1.1	2.5	11.1	2.5	6.5
Mean waist circumference (cm) (SE)	84.2 (15.0)	77.0 (10.1)	78.8 (11.4)	82.1 (12.7)	74.4 (11.9)	77.2 (15.8)
% with increased WC (Men ≥ 90 cm; Women ≥ 80 cm)	31.1	12.2	17.7	57.9	29.7	42.1
Mean blood pressure (SBP/DBP mmHg), (SE)	N-7482 130.6/80.2 (18.9/11.6)	N-6620 126.5/77.4 (18.6/11.6)	N-7529 128.7/79.1 (20.2/12.7)	N-7611 126.4/78.3 (21.6/11.4)	N-6809 123.9/76.6 (20.3/11.4)	N-8022 124.4/77.4 (21.5/11.5)
Grades of high blood pressure (JNC-VII grades) (%)	N- 7510	6639	7551	7630	6829	8033
Normal	23.1	23.7	22.5	19.8	20.1	19.0
Gr I	20.6	15.5	18.1	16.0	13.7	14.3
Gr II	6.6	4.9	6.0	6.4	4.6	5.4
Gr III	3.0	1.9	3.2	3.3	2.3	3.0
Mean fasting blood glucose levels, mg/dl (SE)	83.6 (5.4)	76.3 (5.4)	74.5 (7.2)	85.4 (5.4)	80.0 (7.2)	80.1 (5.4)
Mean fasting total cholesterol, mg/dl (SE)	176.9 (11.5)	161 (11.5)	157 (11.5)	180 (15.3)	169.2 (11.5)	169 (11.5)
Proportion with blood glucose ≥ 126mg/dl, n, (%)	143 (11.4)	78 (6.2)	111 (8.5)	136 (10.3)	74 (5.7)	138 (9.6)
Proportion with total cholesterol ≥ 200 mg/dl, n (%)	397 (31.7)	245 (19.5)	237 (18.1)	431 (32.8)	344 (26.4)	334 (23.4)

SE, standard error; WC, waist circumference; SBP, systolic blood pressure; DBP, diastolic blood pressure

All the centers covered the target sample size of 250 per quinquennial age group among men and women. Smoking forms of tobacco was more common than smokeless tobacco use which was more prevalent in Dibrugarh and Nagpur. Rural and slum areas showed a higher proportion of tobacco users as compared to urban areas. Among adolescents, smoking was most prevalent in urban slums. Many factors like seasonal and regional variation in consumption patterns, fasting or religious beliefs, consumption of tubers and roots, non-capture of casual (non-meal) consumption of fruits, *etc*., influenced the results. There were wide variations in the type of activity in urban and rural areas. Blood pressure was more frequently self-reported to be measured during the past 12 months in the urban men and women (42.1, 52% respectively). Interestingly, 29.8 per cent women from rural areas also reported being measured for their blood pressure (probably as part of the Maternal and Child Health Programmes). Health professionals (doctors and health workers) diagnosed elevated blood pressure in 6–18 per cent of the subjects. More than half of these subjects were currently on some anti-hypertensive medication. It was also found that the respondents found it difficult to differentiate routine physical activity related to daily living versus purposive.

## Scope for surveillance in India

The scope for success of a surveillance programme relies on its sustainability, flexibility, appropriateness of data collected and timely dissemination to its users for action. In India, several reports on CVD risk factors have been brought out in different regions and populations. Many of these are repeated surveys in the same population at random time intervals. There are surveys conducted by various agencies, but the information remains un-utilized for action related to CVD risk factors. These surveys have been able to demonstrate changes in the risk factor profile. Collectively, these have been useful in raising an alarm amongst health planners and policy makers, and for making a case for initiating interventions. Efforts to harmonize these local surveys so as to make them useful for surveillance systems would improve efficiency. It would help in overcoming the limited understanding of surveillance systems amongst health planners and policy makers. Advocacy and dissemination in easy formats will perhaps be required to encourage utilization of data for action at the lowest level of the health system. Building community partnerships would enhance the acceptability and accountability of surveillance data.

In India, several researchers have been able to build networks to carry out CVD related public health activities. Reddy *et al*^32^ reported establishment of a CVD sentinel network amongst 10 industries spread out across various parts of the country. The health behaviours and other CVD risk factors of the employees and their dependants are being monitored periodically. The impact of interventions being carried out is also monitored. The ICMR has established formal links with various governments, universities, institutions, agencies across the world to foster biomedical research. Surveillance for non-communicable diseases has been identified as a priority area with some of them (Canadian Institutes of Health Research, CIHR, University of Minnesota, USA, International Clinical Epidemiology Network, INCLEN). Similar efforts have been brought to notice by various other agencies, institutions and organizations. All these efforts will facilitate sharing of experience internationally.

The ICMR-WHO collaborative initiative on NCD risk factor surveillance (as described earlier) at six sites provided the requisite experience and mandate to respond to the call of the Ministry of Health &; Family Welfare, Government of India, to develop the strategy and modules for undertaking NCD risk factor surveillance at a national level. The World Bank supported the Government of India to conduct the NCD Risk Factor Surveillance under the Integrated Disease Surveillance Project (IDSP) in 29 States/UTs in three phases, beginning in 2007. The ICMR has been identified to co-ordinate, supervise and provide quality assurance to these surveys. The programme has been structured at the national and State level so as to make it integrated and sustainable. The National Nodal agency co-ordinates the surveys through the 5 Regional Research Centers (All India Institute of Medical Sciences, New Delhi; Regional Medical Research Center, Dibrugarh; Shree Chitra Tirunal Institute for Medical and Science Technology, Thiruvananthapuram; Regional Medical Research Center for Tribals, Jabalpur; and National Institute of Epidemiology, Chennai). The State survey agencies are selected to carry out the field data collection. The programme is monitored by a National Technical Advisory Committee and the National Monitoring Committee. Quality assurance mechanisms have been incorporated. The 1^st^ phase of these surveys are being completed in 7 States, and risk factor data on consumption of tobacco, alcohol, fruits and vegetables, physical activity, waist circumference, body mass index, and blood pressure were collected. Through proper sampling design, the survey results will provide State level estimates of the risk factors in the urban and rural populations, in men and women aged 15–64 yr. It is envisaged that after every 3–5 years, these surveys will be repeated at the State level by the respective governments.

In January 2008, the Ministry of Health &; Family Welfare launched the pilot phase of the National Programme for Prevention and Control of Diabetes, CVD and Stroke in 10 districts of the country, and would eventually cover the entire country. The programme aims at providing health promotion, screening for NCDs, setting up specialty clinics, re-orientating the health system towards NCDs, building capacities, and strengthening linkages between various stakeholders. The activities would be aimed at community level, workplace and school settings. The NCD risk factor surveillance being conducted under IDSP has been incorporated as part of the programme. It would collect data, determine priorities, assist planning, evaluate interventions, guide health policies, monitor programme goals, foster research, assess and document in-time needs and serve in developing long-term strategies.

Surveillance can be established at the National, Regional, State and Local levels by linking the data collection activities to policy development and interventions. How can the stakeholders (government, local authorities, public health workers, academicians and researchers) benefit from a partnership exercise? It could be considered as an interaction between the givers and takers, with reversal of roles from time to time. A constant dialogue to assess the needs should be formalized so that surveillance systems can adapt to the requirements. Although the authorities would give the ‘field’ for data collection to the investigators, but in return will expect results, assistance in developing and implementing intervention activities for the population under consideration, *e.g*., the industries would agree to do risk factor surveys, but they will look towards to the researcher for guidance on how and what actions to be taken, so that this becomes a mutually beneficial exercise. The success of this partnership will be reflected in the participation of the community in such programmes. A need for more rapid and advance data collecting tools would be required, such as telephone surveys, e-mail and internet surveys. The use of technology needs to be evaluated against identity protection, costs and validity of information collected. Surveys should be designed in cost effective manner if rapid information is required. Multi-modal methods would require an understanding of local literacy, awareness, cultural contexts, *etc*.

## Conclusions

The burden of CVD and its risk factors in India calls for a sound public health approach to stem the epidemic. Efforts to put in place an intervention programme should be complemented with a robust surveillance mechanism so as to monitor, evaluate and guide policies and programmes. It has been demonstrated in a pilot mode that it is feasible to establish surveillance for CVD risk factors at community levels. It has been scaled up to the national level, and is now included in the National Programme for Prevention and Control of Diabetes, Cardiovascular Diseases and Stroke. The future of surveillance systems lies in its timeliness, systems approach and enduring partnerships. Consolidating on the gains should pave the path for the way forward.
